# Genetic Structuring across Marine Biogeographic Boundaries in Rocky Shore Invertebrates

**DOI:** 10.1371/journal.pone.0101135

**Published:** 2014-07-01

**Authors:** Adriana Villamor, Federica Costantini, Marco Abbiati

**Affiliations:** 1 Department of Biological, Geological and Environmental Sciences, University of Bologna, Ravenna, Emilia-Romagna, Italy; 2 Institute of Marine Sciences, National Research Council, Bologna, Emilia-Romagna, Italy; Simon Fraser University, Canada

## Abstract

Biogeography investigates spatial patterns of species distribution. Discontinuities in species distribution are identified as boundaries between biogeographic areas. Do these boundaries affect genetic connectivity? To address this question, a multifactorial hierarchical sampling design, across three of the major marine biogeographic boundaries in the central Mediterranean Sea (Ligurian-Tyrrhenian, Tyrrhenian-Ionian and Ionian-Adriatic) was carried out. Mitochondrial COI sequence polymorphism of seven species of Mediterranean benthic invertebrates was analysed. Two species showed significant genetic structure across the Tyrrhenian-Ionian boundary, as well as two other species across the Ionian Sea, a previously unknown phylogeographic barrier. The hypothesized barrier in the Ligurian-Tyrrhenian cannot be detected in the genetic structure of the investigated species. Connectivity patterns across species at distances up to 800 km apart confirmed that estimates of pelagic larval dispersal were poor predictors of the genetic structure. The detected genetic discontinuities seem more related to the effect of past historical events, though maintained by present day oceanographic processes. Multivariate statistical tools were used to test the consistency of the patterns across species, providing a conceptual framework for across-species barrier locations and strengths. Additional sequences retrieved from public databases supported our findings. Heterogeneity of phylogeographic patterns shown by the 7 investigated species is relevant to the understanding of the genetic diversity, and carry implications for conservation biology.

## Introduction

Through the study of species distribution as well as patterns of genetic structuring, a link between biogeography and phylogeography can be explored, which aids in the identification of discontinuities in the spatial distribution and genetic diversity of species [1]. The level of connectivity between geographic areas is mainly determined by the interplay between the dispersal capacity of a species (including pre and post-settlement processes [2]), the hydrological regime [3] and the occurrence of geological/topographical boundaries, which may reduce the dispersal potential and hence the gene flow [4].

Major barriers to gene flow between oceanic basins have previously been identified (for example, Point Conception in California, [5]; the Eastern Pacific barrier, [6]; the Indo-Pacific Barrier, [7]; the South-eastern Australian barrier, [8]). These barriers support the occurrence of genetic discontinuities between the world's biogeographical regions [9]. Significant genetic structuring has also been detected at smaller spatial scales in correspondence with the boundaries between biogeographic provinces [10] or sectors [11], [12].

In the Central Mediterranean Sea nine biogeographic sectors have been proposed based on species' distributions [13], but their consistency with phylogeographic sectors has only been investigated in a few cases [14]–[16]. These studies investigated genetic differentiation between the Mediterranean basins (eastern, western, Adriatic Sea) in a single species. However, data from a single species, with specific life history traits, ecology, ethology, etc, cannot be used to generalize phylogeographic barriers or limitations to gene flow across other species. In fact, studies on different target species conducted in the same geographical areas often reveal contrasting genetic structures and/or phylogeographical patterns [17]–[19]. Comparative phylogeography has recently been suggested as a powerful tool to discern general patterns over species-specific traits [20], [21]. It has proven effective in identifying across taxa barriers to gene flow in the Almeria-Oran Front [22], and along the north-eastern Pacific coast [23]. Currently no multispecies studies identifying barriers to gene flow or genetic discontinuities in the Mediterranean Sea exist. Given the practical implications of defining genetic units for the conservation and management of natural resources, a study of this nature is sorely needed [24].

In the present study, the occurrence of phylogeographic boundaries in the Central Mediterranean Sea, and their consistency across species was investigated. By means of a multifactorial hierarchical sampling design, three putative long-term barriers to gene flow were tested. These putative barriers were established based on known biogeographic boundaries [13]. Two replicated areas were sampled across each barrier, and within each area the genetic structure of seven common shallow water invertebrate species was analysed using a portion of the COI mitochondrial gene.

In addition to genetic differentiation measures, two types of multivariate analysis were used to test the consistency of the phylogeographical patterns across species. The results of this study provide a first contribution towards a comparative phylogeography in the Central Mediterranean Sea, with the aim of fully understanding the main past and present drivers of genetic diversity and differentiation.

## Materials and Methods

### Study areas and species

**Figure 1:**
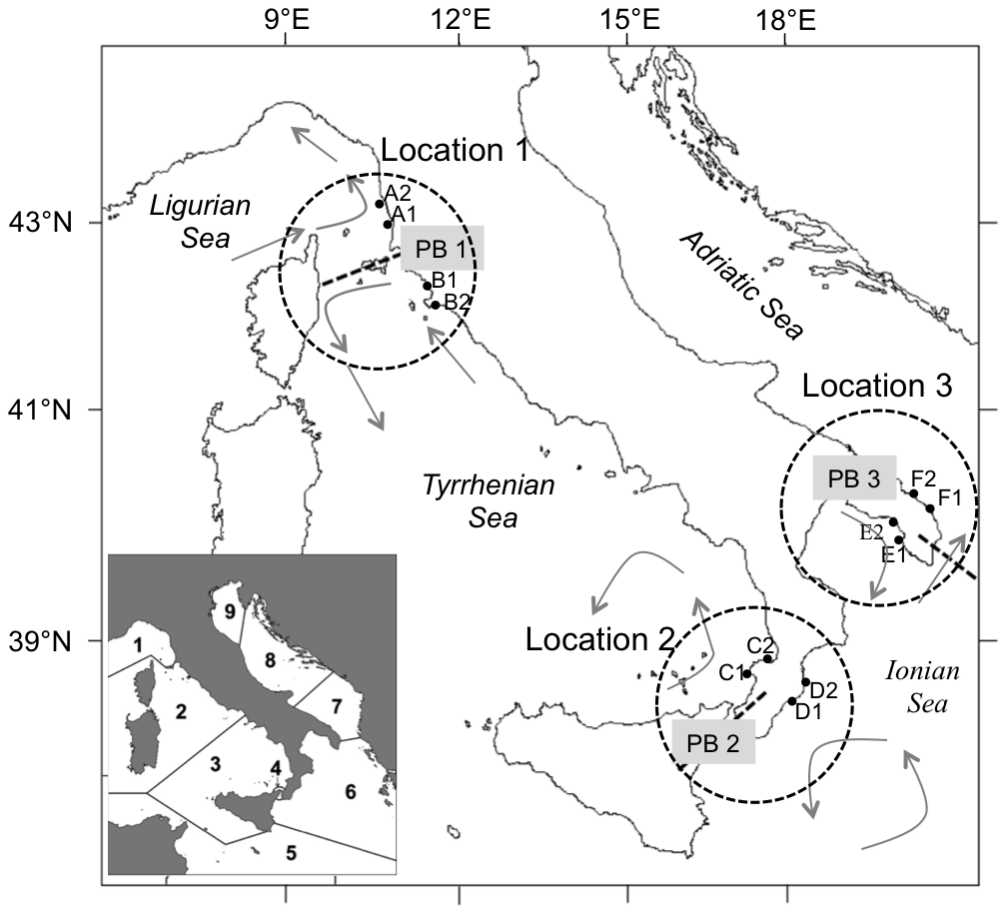
Map of the study area, locations defined, sampling areas and putative barriers analyzed. Location 1: A1. Castiglioncello, A2. Calafuria, B1. Talamone, B2. Porto Ercole; Location 2: C1. Nicotera, C2. Vibo Marina, D1. Soverato, D2. Copanello; and Location 3: E1. Gallipoli, E2. Porto Cesareo, F1. Porto Badisco, and F2. Otranto. PB1: putative barrier between Ligurian and Tyrrhenian Seas, PB2: putative barrier between Tyrrhenian and Ionian Seas and, PB3: putative barrier between Ionian and Adriatic Seas. Grey arrows represent the direction of the dominant currents in those areas. The bottom left image is taken from [65] and shows the most commonly acknowledged biogeographic sectors in the central Mediterranean region.

Three major potential biogeographic/phylogeographic boundaries, located in the Central Mediterranean Sea (Figure 1), and hereafter referred to as putative barriers (PB), were investigated. The first putative barrier (PB1, in Location 1) separates the Ligurian Sea and the North Tyrrhenian Sea, corresponding to a virtual line between the coast of Tuscany, and the islands of Elba and Corsica. The two sides of PB1 are characterized by divergent currents [25]. The second putative barrier (PB2, in Location 2) separates the Tyrrhenian and Ionian Seas, and corresponds to a line across the Strait of Messina and Sicily. Very strong tidal currents characterize the narrow Strait of Messina, flowing mainly from north to south [25]. The third putative barrier (PB3, in Location 3) separates the Ionian and Adriatic Seas, around the Apulian Cape of Santa Maria di Leuca. Strong currents with different patterns in opposite directions characterize the water circulation along this putative barrier [26].

At each side of the putative barriers two replicate sites 150 km apart from each other were defined (sites A, B, C, D, E, F). In each site 2 sampling areas about 25 km apart were randomly selected, and named counter-clockwise starting from Location 1 (areas A1, A2; B1, B2; C1, C2; D1, D2; E1, E2; F1, F2; Figure 1).

**Figure 2:**
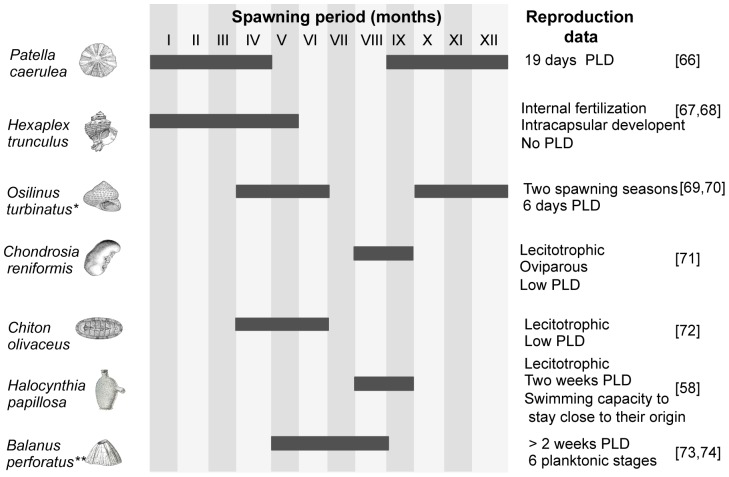
Pelagic larval dispersal estimates, spawning period, and other reproductive data for the species studied. Data retrieved from [58], [66]–[74]. Roman numerals correspond to the months and black bars indicate the extent of the spawning season. * The accepted name of *Osilinus turbinatus* is *Phorcus turbinatus*. ** The accepted name of *Balanus perforatus* is *Perforatus perforatus* (World Register of Marine Species, www.marinespecies.org).

Seven invertebrate species were selected based on their prevalence in the central Mediterranean as well as to cover a variety of life history traits. The selected species belong to different taxa: Gastropoda (3), Polyplacophora (1), Ascidiacea (1); Crustacea (1), and Porifera (1). Data retrieved on life history traits and larval ecology are summarized in Figure 2. The longest Pelagic Larval Duration (PLD) estimates correspond to *Balanus perforatus*, *Patella caerulea*, and *Halocynthia papillosa*. *Hexaplex trunculus* is a brooding species and has no theoretical PLD. PLD estimates for *Chondrosia reniformis*, *Chiton olivaceus*, and *Osilinus turbinatus* are of about one week.

In each area 30 individuals per species were collected, fixed in 80% ethanol and maintained at 4°C prior to processing. Only the sponge was sampled as pieces rather than as whole organism. To minimize the possibility of clonality, samples were collected at least 5 m apart [27]. No specific permits were required for the areas and the species studied.

### DNA extraction and COI amplification

Total DNA was extracted from a piece of circa 5 g of tissue using a REDExtract-N-Amp kit (Sigma–Aldrich). The COI gene was chosen for analysis due to its intermediate rate of variation that provides information on genetic structure across a wide range of spatial and temporal scales [28]. Universal primers described in [29] were initially used for the amplification of a fragment of the COI gene. After the first sequences were retrieved, six pairs of species-specific primers were designed with the software PRIMER v. 3.0 (http://www.fokker.wi.mit.edu/primer3/input.htm) in order to increase amplification success and the quality of sequences obtained (Table S1).

PCR amplification reactions were carried out in a 25 µl total volume with 5 µl of 5x GoTaq Flexi Buffer (Promega), 4 µl of MgCl_2_ 25 mM, 0.5 µl of dNTP mix 10 mM, 0.5 µl of each primer, 0.15 µl of GoTaq DNA polymerase (Promega) and 2.5 µl of template DNA (from a 1∶50 dilution of the extracted DNA). A hold at 94°C for 3 min was followed by 30 cycles of denaturation at 94°C for 45 s, annealing at a primer specific temperature for 45 s and extension at 72°C for 90 s, finishing with a final extension at 72°C for 5 min on a thermal cycler (Applied Biosystems, GeneAmp 2700) (Table S1). PCR products were purified and sequenced by MACROGEN INC.

All sequences were checked and edited manually on Chromas Pro (Technelysium, Pty, Ltd, Australia). Sequence alignments were performed using Mega v. 5 [30].

### Data analysis

Preliminary analysis showed no differentiation in any of the investigated species between the two replicated areas of each site. As a result, individuals from the two areas were pooled and hereafter data have been analysed considering only the sites (A, B, C, D, E, F; Table 1). We detected no genetic differentiation between Locations 1 and 3 of *Balanus perforatus*, 1500 km apart, so decided not to analyse Location 2 samples.

**Table 1:** Number of individuals sequenced (n), haplotypes (h), haplotypic (Hd) and nucleotidic (π) diversity values per location, site and for the whole data set of each species.

Species	Location	Site	n	H_site_	Hd_site_ ± stdev	Π_site_ ± stdev	h_area_	Hd_loc_ ± stdev	Π_loc_± stdev	h_sps_	Hd_sps_ ± stdev	π_ sps_ ± stdev(all values are 10^3^)
*Patella caerulea*	1	A	38	12	0.717±0.078	2.92±0.54	22	0.691±0.057	2.85±0.38	36	0.769±0.022	2.77±0.19
		B	44	13	0.671±0.078	2.78±0.51						
	2	C	28	9	0.730±0.063	2.32±0.39	18	0.820±0.037	2.91±0.31			
		D	27	12	0.863±0.050	3.16±0.43						
	3	E	29	6	0.621±0.086	2.00±0.40	7	0.572±0.060	1.79±0.27			
		F	34	5	0.545±0.085	1.65±0.35						
*Hexaplex trunculus*	1	A	24	5	0.312±0.121	3.82±2.86	6	0.262±0.096	2.66±1.98	27	0.753±0.040	131.7±1.03
		B	12	2	0.167±0.134	0.32±0.26						
	2	C	12	5	0.803±0.078	6.58±3.37	13	0.915±0.031	15.86±1.25			
		D	15	9	0.848±0.088	12.75±3.54						
	3	E	20	6	0.763±0.066	11.21±1.76	11	0.790±0.046	12.64±0.83			
		F	17	8	0.846±0.062	13.86±1.09						
*Osilinus turbinatus*	1	A	30	12	0.766±0.074	2.38±0.42	26	0.772±0.050	3.12±0.43	48	0.788±0.029	3.41±0.31
		B	45	18	0.771±0.063	3.42±0.63						
	2	C	14	6	0.769 0.089	2.33 0.55	14	0.889±0.042	4.18±0.64			
		D	14	9	0.923 0.050	4.89 0.65						
	3	E	45	10	0.737±0.056	3.37±0.67	24	0.888±0.021	4.68±0.55			
		F	31	10	0.789 0.055	4.01 0.91						
*Chondrosia reniformis*	1	A	25	2	0.080±0.072	0.19±0.17	3	0.113±0.072	0.27±0.17	5	0.324±0.055	1.40±0.24
		B	10	2	0.200±0.154	0.47±0.36						
	2	C	13	1	0	0	1	0	0			
		D	13	1	0	0						
	3	E	11	3	0.618±0.104	2.47±0.37	3	0.544±0.036	2.40±0.11			
		F	22	2	0.524±0.033	2.45±0.15						
*Chiton olivaceus*	1	A	25	7	0.430±0.124	0.95±0.31	11	0.442±0.100	1.09±0.29	20	0.554±0.056	1.46±0.20
		B	15	4	0.476±0.155	1.28±0.50						
	2	C	10	4	0.533 0.018	1.19 0.47	4	0.297±0.115	0.62±0.26			
		D	14	3	0.275±0.148	0.57±0.32						
	3	E	27	7	0.609±0.102	3.35±1.23	11	0.775±0.047	2.45±0.34			
		F	16	7	0.808±0.093	3.23±0.64						
*Halocynthia papillosa*	1	A	29	11	0.872±0.036	6.25±0.54	16	0.854±0.030	5.96±0.45	21	0.864±0.019	6.26±0.32
		B	29	8	0.832±0.042	5.58±0.69						
	2	C	10	5	0.844±0.080	7.37±1.02	12	0.926±0.032	8.11±0.51			
		D	12	9	0.939±0.058	8.47±0.15						
*Balanus perforatus*	3	E	15	5	0.790±0.073	4.88±0.102	7	0.767±0.041	4.11±0.53	43	0.882±0.030	5.30±0.48
		F	25	5	0.777±0.043	3.79±0.50						
	1	A	30	19	0.910±0.045	5.68±0.83	34	0.917±0.027	5.34±0.53			
		B	36	21	0.929±0.032	5.12±0.64						
	3	E	16	9	0.767±0.113	4.44±1.13	16	0.795±0.063	5.26±0.96			
		F	17	9	0.831±0.085	5.17±1.37						

### a. Genetic diversity, differentiation and gene flow estimates

Number of haplotypes (h), haplotype and nucleotide diversity (Hd and π, respectively), were calculated for each species by site, location, and for the whole study area, using DnaSP v. 4.50.3 [31]. Diversity values and relationships between the haplotypes were visualized in unrooted haplotype networks, calculated by Median Joining with the software Network v. 4.6.1.1 [32]. Loops were solved when possible following criteria derived from coalescent theory [33].

The fixation index (Fst) between sites across each PB was used to quantify the differentiation due to genetic structure. Large scale genetic structuring across the whole study area was also assessed, as the genetic differentiation between all pairs of sites. Pairwise Fst values were calculated in Arlequin v 3.1 [34] with 10000 permutations, and their significance corrected for multiple testing following [35].

Analysis of Molecular Variance (AMOVA) was performed to investigate sources of variability within and between the three locations studied. Significance was tested with 16000 permutations in Arlequin v 3.1 [34].

Isolation by distance across the whole study area was tested with a Mantel test as implemented in Genepop, following the Isolde procedure which compares a semi matrix of Fst/(1-Fst) with a semi matrix of the natural logarithm of the shortest oceanic distance in km.

Migration rates between areas were estimated with the software MIGRATE-n v. 3.3.1 [36], using Markov chain parameters.

COI sequences of the target species available in Genbank (Table S2) were aligned and represented in haplotype networks with the haplotypes obtained in this study, to provide some insight on large-scale genetic diversity of the species and patterns across other acknowledged barriers to gene flow.

### b. Estimation of effective population size variations

The history of effective population size (Ne) was assessed by testing the fit of the mismatch distributions to the theoretical distribution in an expansion scenario [37] using Arlequin. Fu's and Tajima's neutrality tests were also carried out, as they are more sensitive to population expansions than the mismatch distributions [38]. These tests were performed for each site, for the whole data set, and for undifferentiated groups of sites identified by the previous population differentiation analysis.

For those sites that were not significantly different, and did not deviate from the expansion model, the coalescence time τ (i.e. time since the start of a population expansion) was estimated following the formula T = 2υτ [37], where T is the date of growth or decline in units of mutational time and υ = 2 µk, where k is the number of generations per year and μ is the mutation rate per nucleotide.

For the same undifferentiated sites, past changes in effective population sizes and estimates of expansion time were also inferred by generating Bayesian Skyline Plots (BSPs) using the software BEAST v. 1.7.5 [39]. The best-fit models of nucleotide substitution for each species data set were selected with jModeltest v. 0.1.1 [40] and fed into BEAST. Mutation rate estimates used were: 2.4% MY for molluscs [41], 3.1% MY for barnacles [42], and 2.86% MY for ascidians [43]. Given the absence of molecular clock estimates for sponges, the BSP for this species was not calculated. Confidence intervals for Ne through time were calculated from the posterior probability distributions using TRACER [44].

### c. Multispecies analysis

To test for consistency of the phylogeographic patterns across species, two types of multivariate analysis were used.

The multivariate Analysis of Similarity (ANOSIM), as implemented in Primer v.6 [45], was used to test the differences across each of the three putative barriers. With this analysis we aimed to provide an across-species analysis of haplotype frequencies across the putative barriers, reassuming most of the information on the genetic structure of all species, and minimizing the effect of between species sample size and/or genetic diversity differences. The frequencies of each haplotype of all seven species were the variables, and the Areas were the samples. In order to accomplish with the independence of the variables, the haplotype frequencies were calculated for each haplotype across all sites (instead of for each site across all haplotypes). We used *Site* and *Location* as crossed factors in order to test for the effect of each of the PB. A Bray Curtis similarity matrix between all sites was represented graphically using a Multidimensional Scaling plot (MDS). Apparent groupings across the whole study area were also statistically tested with ANOSIM.

Discriminant analysis of principal components (DAPC), available in the Adegenet package [46] for R (R core team 2012), was also performed. This technique is designed for multivariate genetic data (multi-locus genotypes). It maximizes the variation between groups by first performing a principal component analysis (PCA) on pre-defined groups or populations and then uses the PCA factors as variables for a discriminant analysis (DA), thus ensuring their independence. To convert our sequences into a multivariate dataset, each polymorphic position of each sequence was codified with a four-digit code, corresponding to the four nucleotides. Only the polymorphic sites were considered, creating a “pseudo-genotype” of each specimen. Polymorphic sites across species were counted as null alleles in order to test all species simultaneously. In this case, the number of variables corresponds to the total number of specimens and the samples correspond to the six sites. This analysis allowed us to take into account not only the haplotype frequencies differences, but also how different these haplotypes are, minimizing the effect of the species (although unavoidable due to the different levels of polymorphism, and hence the number of “pseudo-loci” included per species).

## Results

### Genetic diversity and connectivity patterns

A total of 199 haplotypes were deposited in Genbank with accession numbers KF297371 - KF297570. The number of haplotypes discovered ranged between 5 in *Chondrosia reniformis* and 48 in *Osilinus turbinatus. Patella caerulea* and *Balanus perforatus* presented 36 and 43 haplotypes, respectively. *Hexaplex trunculus*, *Chiton olivaceus*, and *Halocynthia papillosa* showed lower number of haplotypes (Table 1). The highest values of nucleotide diversity were found in *H. trunculus*, and *H. papillosa*; the highest haplotype diversity values were found in *B. perforatus* and *H. papillosa*. *C. reniformis* and *C. olivaceus* showed the lowest diversity values (Table 1). *H. trunculus* and *P. caerulea* both showed the highest diversity values at Location 2. *H. trunculus* at Location 3 also showed high diversity values, whereas *P. caerulea* showed higher values at Location 1.

**Figure 3:**
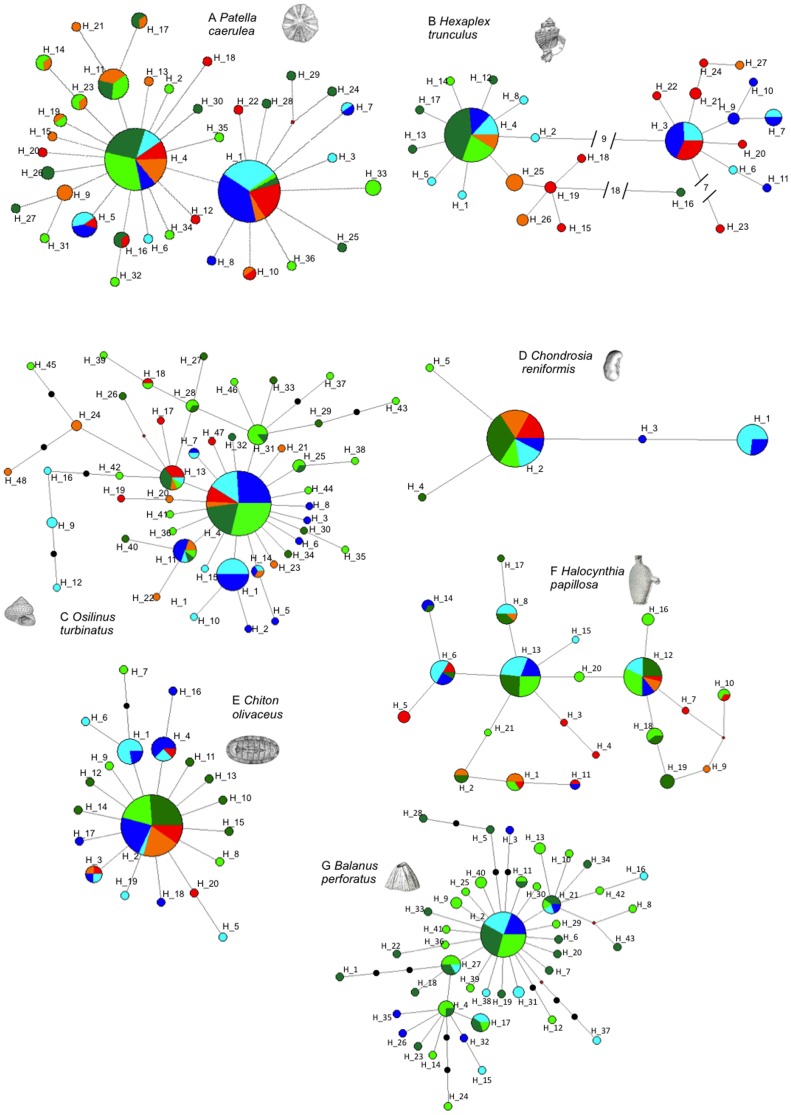
Haplotype networks of single species. Color legends are Location 1 site A: dark green and site B: light green; Location 2 site C: orange and site D: red; Location 3 site E: dark blue and site F: light blue.

*Patella caerulea* and *Hexaplex trunculus* showed two haplogroups in their networks. *P. caerulea* presented two common haplotypes separated by a single mutation and surrounded by several low frequency haplotypes, 26 of which were private (Figure 3a). In *H. trunculus*, nine mutation steps separated the haplogroups, which could therefore be considered as two divergent lineages. The most common lineage occurred across all sites, whereas the second lineage was found in sites D, E, and F (except for H_27, Figure 3b). Two other highly divergent haplotypes were found in site A (H_16) and site E (H_23).

Three species presented star-like shaped haplotype networks with different degrees of ramification. The *Balanus perforatus* network (Figure 3g) showed a high number of low frequency haplotypes. *Osilinus turbinatus* also showed a profusely branched network, with several middle frequency haplotypes (H_1, H_11, H_13, H_31; Figure 3c). The *Chiton olivaceous* network had a simple star shape: one very common haplotype surrounded by several private and three low-frequency haplotypes, with a maximum distance of three mutation steps (Figure 3e).

The five haplotypes of *Chondrosia reniformis* (one common haplotype, three privates, and a middle frequency haplotype in sites E and F, Figure 3d) were separated by a maximum of 2 mutation steps. *Halocynthia papillosa* presented several closely related common haplotypes, and few private haplotypes (11 out of 21, Figure 3f).

Fst values failed to detect significant genetic differentiation across the putative barriers PB1 and PB3 for any of the seven species studied (Table 2). Significant genetic differentiation (p<0.015 after FDR correction) was only detected across the PB2 for *Patella caerulea* and *Hexaplex trunculus* (Table 2a–b). Pairwise Fst values across the whole study area showed significant differences in *P. caerulea* and *H. trunculus* between northwestern (A, B and C) and southeastern sites (D, E and F). *P. caerulea* migration rate estimates were higher from the southeastern towards the northwestern sites (M_N->S_ = 96.8; M_S->N_ = 795.0), whereas *H. trunculus* migration rate estimates were higher from northwestern towards southeastern sites (M_N->S_ = 658.5; M_S->N_ = 25.4). The Mantel test on the geographic and genetic distances matrix was not significant (p = 0.135 for *P. caerulea* and p = 0.082 for *H. trunculus*).

**Table 2:** Pairwise Fst values between sites for all seven species.

	A	B	C.	D	E	F
***a.Patella caerulea***						
A	0					
B	0.00420	0				
C	−0.00626	0.00359	0			
D	**0.07466**	**0.11072**	**0.11252**	0		
E	**0.20938**	**0.25537**	**0.25275**	0.03138	0	
F	**0.16864**	**0.21171**	**0.20695**	0.00932	−0.02537	0
***b. Hexaplex trunculus***						
A	0					
B	−0.02875	0				
C	**0.16035**	**0.22265**	0			
D	**0.67033**	**0.66462**	**0.53127**	0		
E	**0.59243**	**0.58192**	**0.48037**	0.02646	0	
F	**0.37615**	**0.34750**	**0.26302**	0.14552	0.03280	0
***c. Osilinus turbinatus***						
A	0					
B	−0.00414	0				
C	**0.08221**	**0.09829**	0			
D	0.01355	0.04912	0.04474	0		
E	**0.09587**	**0.10261**	**0.16009**	**0.15478**	0	
F	**0.09282**	**0.10546**	**0.10968**	0.08825	0.03916	0
***d. Chondrosia reniformis***						
A	0					
B	0.0318	0				
C	−0.0293	0.0274	0			
D	−0.0293	0.0274	0.0000	0		
E	**0.4632**	0.3062	**0.3838**	**0.3838**	0	
F	**0.4739**	**0.3631**	**0.4078**	**0.4078**	−0.0509	0
***e. Chiton olivaceus***						
A	0					
B	−0.0073	0				
						
C	−0.0136	0.0000	0			
D	0.0071	0.0143	−0.0167	0		
E	**0.0653**	0.0577	0.0512	−0.0172	0	
F	**0.1428**	**0.1309**	**0.1405**	0.0005	0.0118	0
***f. Halocynthia papillosa***						
A	0					
B	0.0152	0				
C	0.1212	0.1414	0			
D	**0.2030**	**0.2225**	0.0890	0		
E	0.0903	**0.1705**	**0.2325**	0,1024	0	
F	0.0897	**0.1716**	**0.2958**	**0.2018**	−0.0336	0
***g. Balanus perforatus***						
A	0					
B	−0.0127	0				
C	-	-				
D	-	-	-	-		
E	−0.0206	−0.0071	-	-	0	
F	−0.0188	−0.0137	-	-	−0.0201	0

Values in bold indicate significance after correction for multiple testing.

In *Osilinus turbinatus* and *Chondrosia reniformis* significant pairwise Fst values were detected between the E and F sites (Location 3) and most other sites (Figure 3c–d), except sites D and F in *O. turbinatus*, and sites B and E in *C. reniformis* (Table 2c–d). Migration rate estimates were bidirectional but asymmetric, higher from sites A, B, C, D, towards sites E and F (*O. turbinatus* M_1+2->3_ = 760.0, M_3->1+2_ = 85.2; *C. reniformis* M_1+2->3_ = 656.1, M_3->1+2_ = 363.0). Mantel tests were not significant in either species (p = 0.136 and 0.101, respectively).

*Chiton olivaceus* showed significant genetic differentiation between sites A, B, C and site F. Site E was also significantly different from site A (Table 2e). A significant Mantel test (p = 0.014) suggests that this pattern of genetic differentiation could be attributed to isolation by distance. Migration estimates were higher between nearby than between distant locations (M_1->2_ = 142.0; MPB_1->3_ = 154.7; M_2->1_ = 353.6; M_2->3_ = 811.6; M_3->1_ = 315.4; M_3->2_ = 302.3).

*Halocynthia papillosa* showed significant genetic differentiation between several pairs of sites (Table 2f,) with no relation to distance (Mantel test p = 0.158). AMOVA analysis on the three locations wasn't significant (p = 0.064; Table S3), however it attributed a high percentage of variation to the differences between them (Va% = 13.56). Migration rate estimates showed wide confidence intervals (data not shown). The acorn barnacle *Balanus perforatus* showed no genetic differentiation between sites across putative barriers (PB1 and PB3), or even across sites located more than 1000 km apart (Table 2g).

Among the 32 sequences of *Patella caerulea* retrieved from Genbank (Table S2), 12 corresponded to the H_4 haplotype (7 from the Western Mediterranean, 2 from Lampedusa, and 3 from west of the Almeria-Oran Front) and six sequences corresponded to the H_1 haplotype (from Greece, Lampedusa, Tuscany, Valencia, and 2 from west AOF). The haplotypes H_11, H_26 and H_17 (Figure 3a, northwestern haplogroup) were found in 6 individuals from the western Mediterranean region, and 4 haplotypes were not found in our dataset. The 11 COI sequences of *Hexaplex trunculus* retrieved belonged to individuals from the South of Portugal (Table S2). Nine of them corresponded to the H_4 haplotype and the other two haplotypes were one nucleotide different from H_4.

Among the 10 *Osilinus turbinatus* COI sequences retrieved (Table S2), 3 (2 from Sardinia and one from SE Spain) presented the H_4 haplotype (Fig. 3c); 3 corresponded to H_9 (Turkey, Cyprus and Croatia); and 2 belonged to H_24 (SE Spain). Two individuals from Croatia and one from Cyprus presented private haplotypes.

The single sequence from *Chondrosia reniformis* published in Genbank corresponded to the H_2 haplotype and belonged to an individual from Israel (Fig. 3d). The *Chiton olivaceus* sequence came from an individual from north-eastern Spain and presented the H_2 haplotype (Fig 3e). The six sequences of *Halocynthia papillosa* (Table S2) belonged to individuals from the north-western region of the Mediterranean. Two of these presented the H_12 haplotype, while the other two presented the H_13 haplotype and the last two showed the H_18 haplotype (Fig. 3f).

### Estimation of population size variations

In four species, mismatch distributions and neutrality tests indicated expansion in most sites, as well as in the whole study area (Figure S1; Table S4). The brooding gastropod *Hexaplex trunculus* did not show signs of expansion in most sites (Table S4b), probably due to the admixture of two genetic pools, visible in the mismatch distribution (Figure S1b) and previously detected as the divergent lineages in the haplotype network. The solitary ascidian *Halocynthia papillosa* and the sponge *Chondrosia reniformis* did not show signs of expansion (Table S4).

Bayesian skyline plots (BSP) showed large confidence intervals in most cases (Figure S2), yet they roughly reflected the estimates of time since expansion calculated with Rogers & Harpending's formula. The northern haplogroup of *Patella caerulea* and the southern haplogroup of *Hexaplex trunculus* demonstrated stable population sizes (Ne) through time, coinciding with estimates of times since expansion (43,600 and 68,200 years ago, respectively) largely preceding the Last Glacial Maximum (LGM: 24,000–27,000 years ago). The more recent lineages showed a slightly growing population size in the BSP (Figure S2), but still dating before the LGM: 29,700 years ago in the southern haplogroup of *P. caerulea* and 31,700 ya in the northern lineage of *H. trunculus*. The BSP of *Balanus perforatus*, *Osilinus turbinatus*, and *Chiton olivaceus* showed increasing Ne through time consistent with the strong signs of expansion detected. Estimates of time since expansion were in accordance with an old expansion for *B. perforatus* (62,600 ya), and coincided with the LGM for *O. turbinatus* (25,300 ya). Expansion estimates for *C. olivaceous* dated around 32,600 ya, before the LGM, in contrast with the BSP, which showed increasing population size after the LGM.

### Multispecies analysis

**Figure 4:**
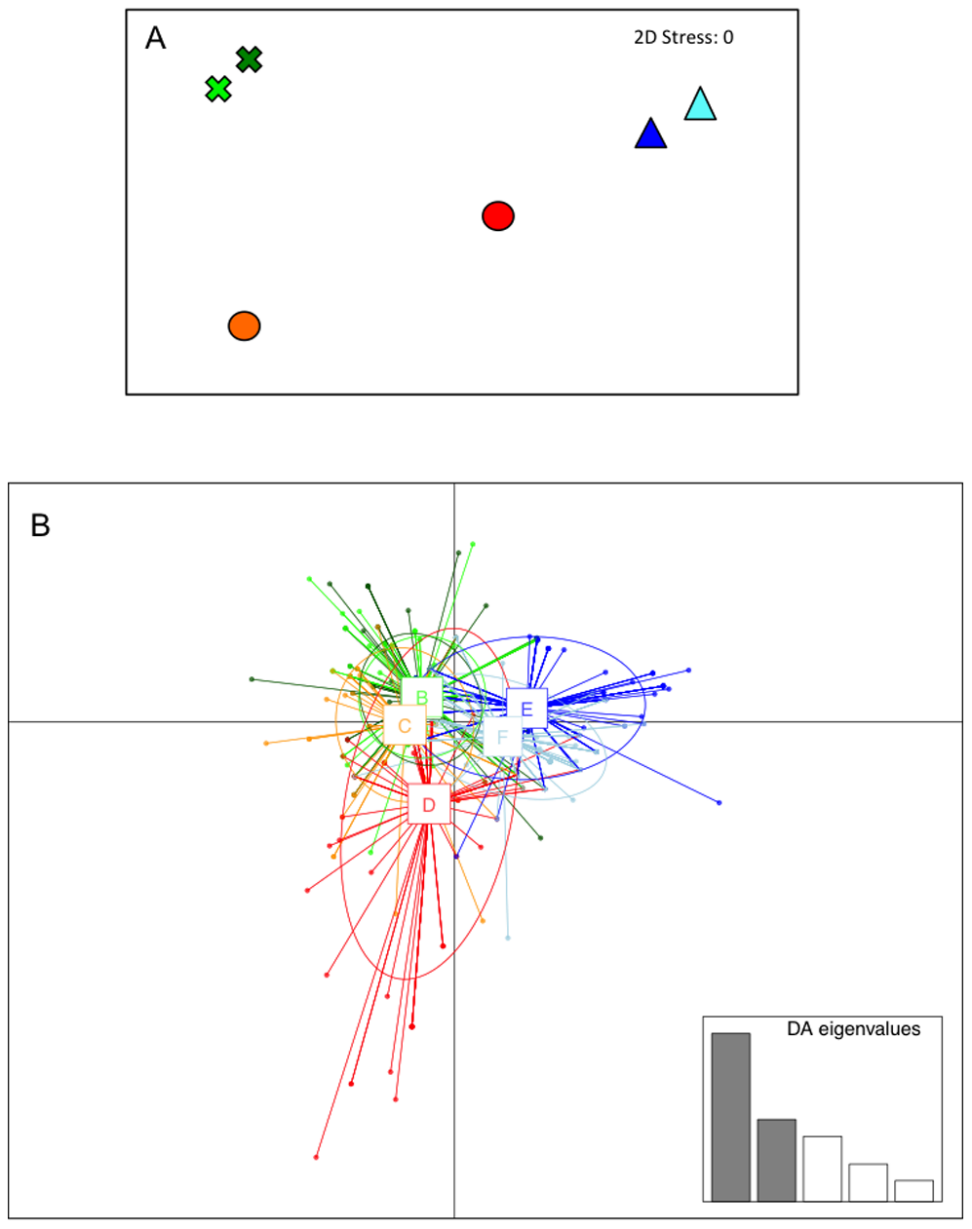
Graphic representation of the multivariate analyses. **a.** MDS of the Bray-Curtis distance matrix of sites according to the distribution of haplotype frequencies across all species, **b.** Graphic result of DAPC. Colour codes are the same as in Figure 3.

A matrix of 159 different variables corresponding to the frequencies of the haplotypes of all species (excluding *Balanus perforatus*) across sampling areas was generated. The analysis of similarity (ANOSIM) indicated no significant effect of the factor site crossed by location, i.e. no multispecies significant differences across any of the three putative barriers. The MDS of the Bray-Curtis matrix of distances between all six sites (Table 3) showed higher similarity of sites A, B (Location 1) and C (Location 2). Site D (Location 2) was isolated, and sites E and F (Location 3) clustered together (Figure 4a). ANOSIM on these three groups showed significant differences between them (R = 1; p = 0.017).

**Table 3:** Bray-Curtis distance matrix between sites according to haplotype frequency distribution across all species.

	A	B	C	D	E	F
A	-					
B	54.93	-				
C	41.95	42.55	-			
D	30.96	32.69	35.41	-		
E	34.97	32.12	32.85	41.38	-	
F	31.76	29.93	30.51	39.34	64.12	-

After transforming haplotypes into pseudo-genotypes, we obtained a matrix of 802 individuals with genotypes of 152 loci each (most of them zeros), corresponding to all polymorphic sites across all species (33 of *Patella caerulea*, 50 of *Hexaplex trunculus*, 8 of *Halocynthia papillosa*, 37 of *Osilinus turbinatus*, 19 of *Chiton olivaceus*, and 4 of *Chondrosia reniformis*). The resulting DAPC showed a distribution of the samples (sites) similar to the MDS plot: sites A, B and C grouped together. Site D was separated from all the others, and sites E and F clustered together (Figure 4b).

## Discussion

In the study area two zones of genetic discontinuity have been identified: across the biogeographic boundary between the Tyrrhenian and Ionian Seas, and between the western and eastern coasts of the Ionian Sea.

The strongest genetic structure detected was across the Tyrrhenian-Ionian biogeographic boundary. The presence of this long-term boundary was reflected in the genetic structure of two species out of the seven studied. It probably corresponds to the boundary between biogeographic sectors 3 and 6 as defined by [13] (see Figure 1). The genetic structure across these sectors has been described by [47]–[49], but the location of the boundary remained vague. In this area, due to the complex topography of straits and islands, Pleistocene sea level fluctuations have left a signature in the genetic structure of many species [48], subjected to periodic divergences and reconnections of the populations on the two sides of the Messina Strait. Past history events might explain the origin of haplogroups or divergent lineages, whereas present day events, such as the current regimes, aid in maintaining and enhancing the differences due to limited or asymmetric connectivity between sites.

*Patella caerulea* showed significant genetic structure across this Tyrrhenian-Ionian PB, reflected in the two haplogroups with different frequencies across each side of the boundary. In spite of the significant differentiation, a bidirectional but asymmetric migration across the barrier was estimated. Other limpet species showed comparable genetic structure in this area [50]. In addition, the sequences retrieved in Genbank gave further support to this structure: western and eastern Mediterranean samples clustered more often with haplotypes of the “northern haplogroup”, and “southern haplogroup”, respectively. It is noteworthy that individuals from the Atlantic side of the Almeria-Oran Front (AOF), the best-known barrier to gene flow in the Mediterranean [20], presented common haplotypes, suggesting this barrier had no large effect on *P. caerulea* genetic structure [51]. Expansion estimates indicate long-term persistence of this species and, in accordance with the older expansion estimates, an older origin of the northwestern haplogroup. This is in agreement with the widespread distribution and abundance of this species and with the more probable Atlantic origin of this genus [52].

The subtidal gastropod *Hexaplex trunculus*, a brooding species, showed the sharpest genetic structure across the Tyrrhenian-Ionian biogeographic boundary. Despite this fact, several haplotypes were common to sites located up to 1500 km apart, on both sides of the putative barrier, probably indicating high migration rates from the Ligurian-Tyrrhenian towards the Ionian-Adriatic populations, but almost none in the opposite direction. This sharp asymmetry has been described in other regions and species, and is suggested to reflect persistent current regimes affecting larval dispersal during the reproductive period [53]. Estimates of expansion of both lineages are consistent with the persistence of this species during the LGM, in more than one glacial refuge, followed by a unidirectional secondary contact [54]. Expansion estimates are older in the southern lineage, which, together with the higher diversity values in this area, indicates a major contribution of the Eastern Mediterranean genetic pool to the total genetic diversity of this species [41]. All sequences retrieved from Genbank belonged to individuals from South Portugal, so no further details on the structure of other Mediterranean sites could be inferred. Most individuals showed haplotypes of the northern haplogroup, reinforcing their Mediterranean origin, but also suggesting no effect of the AOF on the long-term genetic structure of this species, as was the case for *Patella caerulea*.

An unexpected area of genetic discontinuity was disclosed between the eastern and western Ionian Sea. Two species, presumably not affected by the Tyrrhenian-Ionian boundary previously described, showed significant genetic structure across the Ionian Sea, i.e. between the Adriatic-Ionian sites and the rest of the study area. The Ionian Sea has never been considered to host phylogeographic barriers, either related to historical events nor according to present day oceanographic processes. No records referring to genetic structure across this region were found in the literature, despite the fact that the genetic structures of some species support this finding [48], although no explicit tests to identify the barrier had been carried out. The location of the biogeographic boundary separating the eastern and western Mediterranean is controversial [55]. This newly detected phylogeographic boundary provides new insight to understanding the biogeography of the area, although further data on the genetic structure of other species in the area would be needed.

*Osilinus turbinatus* presented a high diversity of haplotypes and significant structure across the Ionian Sea, with bidirectional and symmetric migration estimates. Published sequences supported the detected structure and individuals from the eastern Mediterranean and the Adriatic Sea presented haplotypes related to those found exclusively in the Adriatic-Ionian sites. The estimates of expansion of this species coincide with the Last Glacial Maximum (LGM between 24,000 and 27,000 ya, [20]).

The sponge *Chondrosia reniformis*, in spite of evincing low diversity values, also showed significant genetic structure across the Ionian Sea. A similar differentiation has been described in other widespread sponge species in the same geographical area [56]. The only sequence retrieved from Genbank belongs to an eastern Mediterranean individual and shares the more common haplotype. Although further data from the Ionian Sea and the eastern Mediterranean would be needed to confirm our finding, this study suggests that for *Osilinus turbinatus* and *C. reniformis* the phylogeographic barrier between eastern and western Mediterranean is located in the Mid Ionian Sea.

Significant genetic structure was detected across the northern and southern Tyrrhenian Sea in *Hexaplex trunculus* and *Osilinus turbinatus*. In both cases significant Fst values were likely due to the occurrence of several private haplotypes, south Tyrrhenian haplotypes in the case of *H. trunculus*, and Ligurian-North Tyrrhenian private haplotypes in the case of *O. turbinatus*.

*Halocynthia papillosa* showed several significant pairwise differences between sites across the three locations. The positive values of the neutrality tests, the presence of several common haplotypes, the smaller number of private ones, and the flat shape of the BSP, strongly indicate a long-term stability of the population size. This species may have persisted in two or more glacial refugia, followed by a secondary contact between them [57]. Although this ascidian has large dispersal capacity estimates [43] our results support larval retention, as has been previously suggested ([58], Figure 2), allowing genetic structuring in the range of 300 and 1500 km.

*Balanus perforatus*, the species with the longest PLD estimates, lacked significant genetic structure across distances of up to 1500 km. Neutrality tests indicated a very old expansion, starting around 60,000 ya. This pattern of persistence is similar to that shown by other widespread and abundant species inhabiting comparable habitats along the northeastern Pacific [59], [60].

The genetic structure found among populations of *Chiton olivaceus* across the whole study area is congruent with a pattern of isolation by distance. This species showed the only estimate of expansion time clearly posterior to the LGM, indicating reduced persistence and recent colonization from glacial refugia [60]. This would also be in agreement with the low abundances and low molecular diversities detected with respect to the other species studied, and to other polyplacophora species [3].

As previously shown [61], our results support the weak correlation between PLD and genetic structure. The scale of larval dispersal is related to the complex interaction of larval life history traits, oceanographic regimes, habitat quality and distribution, and the variability of each of these factors through time. However, it is noteworthy that contrasting dispersal capacity patterns, as those of *Hexaplex trunculus* and *Balanus perforatus*, influence connectivity across the boundaries of biogeographic areas, as already detected in fishes [62]. The genetic structure observed is most likely due to the different effects of glacial and interglacial isolations and reconnections on their populations. Levels of gene flow are not the only factors determining present day genetic structure among populations; divergence times and population size can greatly affect the observed genetic structure [63], however the lack of basic biological data for most of our focus species, does not allow to disentangle the role these different drivers play in the observed genetic structures.

The two multivariate analyses allowed a general picture to be provided by merging in a common framework the results obtained for individual species. The statistical tests as well as the graphical representation provided a general picture of the phylogeographic structure in the Central Mediterranean, as well as the relative importance of the discontinuity areas detected, according to the number of species affected. This approach enables to summarize the available genetic information in a simple, straightforward result, which might be useful for decision-making or management purposes. Species with high haplotype and/or nucleotide diversities could bias these analyses; however the multispecies approach counteracts this effect, so the results are not obscured by the structure of the most variable species.

According to the results of the present study, the Tyrrhenian-Ionian biogeographical boundary acts as a phylogeographical barrier, as suggested by the genetic structure of two out of seven invertebrate species. The hierarchical sampling design adopted allowed the identification of an area of genetic discontinuity in the mid-Ionian Sea, reflected in the genetic structure of two species. The data retrieved from public databases reinforced the observed haplotype distributions, and gave further evidence on the variability of the effect of known barriers to gene flow on the genetic structure of different species. In the Mediterranean Sea, Pleistocene glaciations had a profound influence on the present day distribution of species and genetic diversity. The estimates of expansion and divergence allowed further understanding of the genetic structure found at present. Due to the ecological importance of the Central Mediterranean Sea as a hot spot of marine biodiversity [64], these results are of importance for the better understanding of the ecological functioning of its coastal ecosystem. General patterns pertinent to the management and conservation of this important ecosystem have been shown thanks to the comparative phylogeography approach used. Further research analyzing the genetic structure of other species, inclusive of additional Putative Boundaries, while also utilizing a larger set of molecular markers, would provide more insight on a multi-species, multi-scale picture of connectivity in the marine realm.

## Supporting Information

Figure S1 **Mismatch distributions at each Location and for the whole data set for each of the seven species.** Grey lines represent the expected and black lines the observed distribution.
(TIF)

Figure S2 **Bayesian Skyline Plots for the species showing signs of expansion according to mismatch distribution or neutrality tests.** *Patella caerulea* and *Hexaplex trunculus* plots are calculated for each haplogroup. When shown, the black square indicates the date of the Last Glacial Maximum (LGM). The most likely evolutionary model selected by jModelTest is indicated in each plot.
(TIF)

Table S1 **Specific primers.** Names, sequence, amplified fragment length, and optimal annealing temperature for each of the seven pairs of primers used.
(DOCX)

Table S2 **Previously published sequences.** Accession numbers, geographical origin and identification with the haplotypes found in this study, as named in Figure 3, of sequences retrieved from Genbank.
(DOCX)

Table S3 **Analyses of Molecular Variance.** Results of the Amova on the groups corresponding to the three Locations.
(DOCX)

Table S4 **Demographic history analyses.** Fit to sudden expansion model and neutrality tests.
(DOCX)

## References

[1] Slatkin M (1987) Gene flow and the geographic structure of natural populations. Science 236: 787–792. 3576198 10.1126/science.3576198

[2] Cowen RK, Sponaugle S (2009) Larval dispersal and marine population connectivity. Ann Rev Mar Sci 1: 443–466. 10.1146/annurev.marine.010908.163757 21141044

[3] Ayre DJ, Minchinton T E, Perrin C (2009) Does life history predict past and current connectivity for rocky intertidal invertebrates across a marine biogeographic barrier?. Mol Ecol 18: 1887–1903. 19434808 10.1111/j.1365-294x.2009.04127.x

[4] Blanco-Bercial L Alvarez-Marques F, Bucklin A (2011) Comparative phylogeography and connectivity of sibling species of the marine copepod *Clausocalanus* (Calanoida). J Exp Mar Biol Ecol 404: 108–115.

[5] Wares JP, Cunningham CW (2001) Phylogeography and historical ecology of the North Atlantic intertidal. Evolution 55: 2455–2469. 11831661 10.1111/j.0014-3820.2001.tb00760.x

[6] Lessios HA, Robertson DR (2006) Crossing the impassable: genetic connections in 20 reef fishes across the eastern Pacific barrier. P Roy Soc B-Biol Sci 273: 2201–2208. 10.1098/rspb.2006.3543 PMC1635520 16901840

[7] Gaither MR, Toonen RJ, Robertson DR, Planes S, Bowen BW (2010) Genetic evaluation of marine biogeographical barriers: perspectives from two widespread Indo-Pacific snappers (*Lutjanus kasmira* and *Lutjanus fulvus*). J Biogeogr 37: 133–147.

[8] Coleman MA Roughan M, Macdonald HS, Connell SD, Gillanders BM, et al (2011) Variation in the strength of continental boundary currents determines continent-wide connectivity in kelp. J Ecol 99: 1026–1032.

[9] Teske PR, Papadopoulos I, Mmonwa KL, Matumba TG, McQuaid CD, et al (2011) Climate-driven genetic divergence of limpets with different life histories across a southeast African marine biogeographic disjunction: different processes, same outcome. Mol Ecol 20: 5025–5041. 22017655 10.1111/j.1365-294X.2011.05307.x

[10] Luttikhuizen PC, Campos J, van Bleijswijk J, Peijnenburg KTC, van der Veer HW (2008) Phylogeography of the common shrimp, Crangon crangon (L.) across its distribution range. Mol Phylogenet Evol 46: 1015–1030. 18207428 10.1016/j.ympev.2007.11.011

[11] Bird CE, Holland BS, Bowen BW, Toonen RJ (2007) Contrasting phylogeography in three endemic Hawaiian limpets (*Cellana spp*.) with similar life histories. Mol Ecol 16: 3173–3186. 17651195 10.1111/j.1365-294X.2007.03385.x

[12] Drew JA, Barber PH (2012) Comparative phylogeography in fijian coral reef fishes: a multi-taxa approach towards marine reserve design. PLoS One 7: e47710. 23118892 10.1371/journal.pone.0047710 PMC3484158

[13] Bianchi CN, Morri C (2000) Marine biodiversity of the Mediterranean Sea: Situation, problems and prospects for future research. Mar Poll Bull 40: 367–376.

[14] Stefanni S, Thorley JL (2003) Mitochondrial DNA phylogeography reveals the existence of an Evolutionarily Significant Unit of the sand goby *Pomatoschistus minutus* in the Adriatic (Eastern Mediterranean). Mol Phylogenet Evol 28: 601–609. 12927142 10.1016/s1055-7903(03)00054-x

[15] Domingues VS, Bucciarelli G, Almada VC, Bernardi G (2005) Historical colonization and demography of the Mediterranean damselfish, *Chromis chromis* . Mol Ecol 14: 4051–4063. 16262858 10.1111/j.1365-294X.2005.02723.x

[16] Maggio T Lo Brutto S, Garoia F, Tinti F, Arculeo M (2009) Microsatellite analysis of red mullet *Mullus barbatus* (Perciformes, Mullidae) reveals the isolation of the Adriatic Basin in the Mediterranean Sea. ICES J Mar Sci 66: 1883–1891.

[17] Perez-Losada M, Nolte MJ, Crandall KA, Shaw PW (2007) Testing hypotheses of population structuring in the Northeast Atlantic Ocean and Mediterranean Sea using the common cuttlefish *Sepia officinalis* . Mol Ecol 16: 2667–2679. 17594438 10.1111/j.1365-294X.2007.03333.x

[18] Rock J, Ironside J, Potter T, Whiteley NM, Lunt DH (2007) Phylogeography and environmental diversification of a highly adaptable marine amphipod, *Gammarus duebeni* . Heredity 99: 102–111. 17426729 10.1038/sj.hdy.6800971

[19] Papetti C, Martin-Pujolar J, Mezzavilla M, La Mesa M, Rock J, et al (2012) Population genetic structure and gene flow patterns between populations of the Antarctic icefish *Chionodraco rastrospinosus* . J Biogeogr 39: 1361–1372.

[20] Patarnello T, Volckaert FAMJ, Castilho R (2007) Pillars of Hercules: is the Atlantic-Mediterranean transition a phylogeographical break? Mol Ecol 16: 4426–4444. 17908222 10.1111/j.1365-294X.2007.03477.x

[21] Marske KA, Rahbek C, Nogues-Bravo D (2013) Phylogeography: spanning the ecology-evolution continuum. Ecography 36: 1169–1181.

[22] Galarza JA, Carreras-Carbonell J, Macpherson E, Pascual M, Roques S, et al (2009) The influence of oceanographic fronts and early-life-history traits on connectivity among littoral fish species. Proc Natl Acad Sci USA 106: 1473–1478. 19164518 10.1073/pnas.0806804106 PMC2629446

[23] Kelly RP, Palumbi SR (2010) Genetic structure among 50 species of the northeastern Pacific rocky intertidal community. PLoS One 5: e8594. 20062807 10.1371/journal.pone.0008594 PMC2799524

[24] Marti-Puig P, Costantini F, Rugiu L, Ponti M, Abbiati M (2013) Patterns of genetic connectivity in invertebrates of temperate MPA networks. Adv Oceanogr Limnol 4: 138–149.

[25] Millot C (1999) Circulation in the western Mediterranean Sea. J Marine Syst 20: 423–442.

[26] Astraldi M, Balopoulos S, Candela J, Font J, Gacic M, et al (1999) The role of straits and channels in understanding the characteristics of Mediterranean circulation. Prog Oceanogr 44: 65–108.

[27] Duran S, Giribet G, Turón X (2004) Phylogeographical history of the sponge *Crambe crambe* (Porifera, Poecilosclerida): range expansion and recent invasion of the Macaronesian islands from the Mediterranean Sea. Mol Ecol 13: 109–122. 14653793 10.1046/j.1365-294x.2003.02022.x

[28] Avise JC (2000). Phylogeography: the history and formation of species. Harvard University Press.

[29] Folmer O, Black M, Hoeh W, Lutz R, Vrijenhoek R (1994) DNA primers for amplification of mitochondrial cytochrome c oxidase subunit I from diverse metazoan invertebrates. Mol Mar Biol Biotech 3: 294–299. 7881515

[30] Tamura K, Peterson D, Peterson N, Stecher G, Nei M, Kumar S (2011) MEGA5: Molecular evolutionary genetics analysis using Maximum likelihood, evolutionary distance, and maximum parsimony methods. Mol Biol Evol 28: 2731–273. 21546353 10.1093/molbev/msr121 PMC3203626

[31] Rozas J, Sanchez-DelBarrio JC, Messeguer X, Rozas R (2003) DnaSP, DNA polymorphism analyses by the coalescent and other methods. Bioinformatics 19: 2496–2497. 14668244 10.1093/bioinformatics/btg359

[32] Bandelt HJ, Forster P, Rohl A (1999) Median-joining networks for inferring intraspecific phylogenies. Mol Biol Evol 16: 37–48. 10331250 10.1093/oxfordjournals.molbev.a026036

[33] Templeton AR, Boerwinkle E, Sing CF (1987) A cladistic analysis of phenotypic associations with haplotypes inferred from restriction endonuclease mapping. 1. Basic theory and an analysis of alcohol-dehydrogenase activity in *Drosophila* . Genetics 117: 343–351. 2822535 10.1093/genetics/117.2.343 PMC1203209

[34] Excoffier L, Lischer HEL (2010) Arlequin suite ver 3.5: a new series of programs to perform population genetics analyses under Linux and Windows. Mol Ecol Res 10: 564–567. 10.1111/j.1755-0998.2010.02847.x 21565059

[35] Narum SR (2006) Beyond Bonferroni: Less conservative analyses for conservation genetics. Conserv Genet 7: 783–787.

[36] Beerli P, Felsenstein J (2001) Maximum likelihood estimation of a migration matrix and effective population sizes in n subpopulations by using a coalescent approach. P Natl Acad Sci USA 98: 4563–4568. 10.1073/pnas.081068098 PMC31874 11287657

[37] Rogers AR, Harpending H (1992) Population growth makes waves in the distribution of pairwise genetic differences. Mol Biol Evol 9: 552–569. 1316531 10.1093/oxfordjournals.molbev.a040727

[38] Fu YX (1997) Statistical tests of neutrality of mutations against population growth, hitchhiking and background selection. Genetics 147: 915–925. 9335623 10.1093/genetics/147.2.915 PMC1208208

[39] Drummond AJ, Rambaut A (2007) BEAST: Bayesian evolutionary analysis by sampling trees. BMC Evol. Biol. 7: 214. 10.1186/1471-2148-7-214 PMC2247476 17996036

[40] Posada D (2008) jModelTest: Phylogenetic model averaging. Mol Biol Evol 25: 1253–1256. 18397919 10.1093/molbev/msn083

[41] Hellberg ME, Balch DP, Roy K (2001) Climate-driven range expansion and morphological evolution in a marine gastropod. Science 292: 1707–1710. 11387473 10.1126/science.1060102

[42] Campo D, Molares J, Garcia L, Fernandez-Rueda P, Garcia-Gonzalez C, Garcia-Vazquez E (2010) Phylogeography of the European stalked barnacle (*Pollicipes pollicipes*): identification of glacial refugia. Mar Biol 157: 147–156.

[43] Kim WJ, Lee CI, Kim HS, Han HS, Jee YJ, et al (2012) Population genetic structure and phylogeography of the ascidian, *Halocynthia roretzi*, along the coasts of Korea and Japan, inferred from mitochondrial DNA sequence analysis. Biochem Syst Ecol 44: 128–135.

[44] Rambaut A, Drummond AJ (2007) Tracer v1.4. Available from http://beast.bio.ed.ac.uk/Tracer.

[45] Clarke KR, Warwick RM (2001) Change in marine communities: an approach to statistical analysis and interpretation, 2nd edition. PRIMER-E: Plymouth.

[46] Jombart T (2008) Adegenet: a R package for the multivariate analysis of genetic markers. Bioinformatics 24: 1403–1405. 18397895 10.1093/bioinformatics/btn129

[47] Virgilio M, Fauvelot C, Costantini F, Abbiati M, Backeljau T (2009) Phylogeography of the common ragworm *Hediste diversicolor* (Polychaeta: Nereididae) reveals cryptic diversity and multiple colonization events across its distribution. Mol Ecol 18: 1980–1994. 19344353 10.1111/j.1365-294X.2009.04170.x

[48] Serra IA, Innocenti AM, Di Maida G, Calvo S, Migliaccio M, et al (2010) Genetic structure in the Mediterranean seagrass *Posidonia oceanica*: disentangling past vicariance events from contemporary patterns of gene flow. Mol Ecol 19: 557–568. 20051010 10.1111/j.1365-294X.2009.04462.x

[49] Gharbi A, Said K (2011) Genetic variation and population structure of *Holothuria polii* from the eastern and western Mediterranean coasts in Tunisia. J Mar Biol Assoc UK 91: 1599–1606.

[50] Sa-Pinto A, Branco MS, Alexandrino PB, Fontaine MC, Baird SJE (2012) Barriers to gene flow in the marine environment: insights from two common intertidal limpet species of the Atlantic and Mediterranean. PLoS One 7.. 10.1371/journal.pone.0050330 PMC3519802 23239977

[51] Côrte-Real HBSM, Hawkins SJ, Thorpe JP (1996) Population differentiation and taxonomic status of the exploited limpet *Patella candei* in the Macaronesian islands (Azores, Madeira, Canaries). Mar Biol 125: 141–152.

[52] Sa-Pinto A, Branco MS, Harris DJ, Alexandrino P (2005) Phylogeny and phylogeography of the genus *Patella* based on mitochondrial DNA sequence data. J Exp Mar Biol Ecol 325: 95–110.

[53] Collins CJ, Fraser CI, Ashcroft A, Waters JM (2010) Asymmetric dispersal of southern bull-kelp (*Durvillaea antarctica*) adults in coastal New Zealand: testing an oceanographic hypothesis. Mol Ecol 19: 4572–4580. 20875065 10.1111/j.1365-294X.2010.04842.x

[54] Perez-Portela R, Villamor A, Almada V (2010) Phylogeography of the sea star *Marthasterias glacialis* (Asteroidea, Echinodermata): deep genetic divergence between mitochondrial lineages in the north-western mediterranean. Mar Biol 157: 2015–2028.

[55] Pérès JM, Picard JN (1964) Nouveau manuel de bionomie benthique de la Mer Mediterranee. Recueilles des Travaux de la Station marine Endoume 31: 1–137.

[56] Xavier JR, Rachello-Dolmen PG, Parra-Velandia F, Schoenberg CHL, Breeuwer JAJ, van Soest RWM (2010) Molecular evidence of cryptic speciation in the “cosmopolitan” excavating sponge *Cliona celata* (Porifera, Clionaidae). Mol Phylogenet Evol 56: 13–20. 20363344 10.1016/j.ympev.2010.03.030

[57] Maggs CA, Castilho R, Foltz D, Henzler C, Jolly MT, et al (2008) Evaluating signatures of glacial refugia for north Atlantic benthic marine taxa. Ecology 89: S108–S122. 19097488 10.1890/08-0257.1

[58] Santic M, Rada B, Paladin A, Pleslic G (2010) The influence of some abiotic parameters on growth inclination in ascidian *Halocynthia papillosa* (Linnaeus, 1767) from the Northern Adriatic Sea (Croatia). Arch Biol Sci 62: 1007–1011.

[59] Sotka EE, Wares JP, Barth JA, Grosberg RK, Palumbi SR (2004) Strong genetic clines and geographical variation in gene flow in the rocky intertidal barnacle *Balanus glandula* . Mol Ecol 13: 2143–2156. 15245390 10.1111/j.1365-294X.2004.02225.x

[60] Marko PB, Hoffman JM, Emme SA, Mcgovern TM, Keever CC, et al (2010) The ‘Expansion-Contraction’ model of Pleistocene biogeography: rocky shores suffer a sea change? Mol Ecol 19: 146–169. 20092033 10.1111/j.1365-294x.2009.04417.x

[61] Weersing K, Toonen RJ (2009) Population genetics, larval dispersal, and connectivity in marine systems. Mar Ecol-Prog Ser 393: 1–12.

[62] Riginos C, Douglas KE, Jin Y, Shanahan DF, Treml EA (2011) Effects of geography and life history traits on genetic differentiation in benthic marine fishes. Ecography 34: 566–575.

[63] Marko PB, Hart MW (2011) The complex analytical landscape of gene flow inference. TREE 26: 448–456. 21722987 10.1016/j.tree.2011.05.007

[64] Coll M Piroddi C, Steenbeek J, Kaschner K, Ben Rais Lasram F, et al (2010) The biodiversity of the Mediterranean Sea: estimates, patterns, and threats. PLoS One 5: e11842. 20689844 10.1371/journal.pone.0011842 PMC2914016

[65] Bianchi CN (2004) Proposta di suddivisione dei mari italiani in settori biogeografici. Notiziario SIBM 46: 57–59.

[66] Dodd JM (1957) Artificial fertilisation, larval development and metamorphosis in *Patella vulgata* L. and Patella caerulea L. Mar Ecol PSZN 29: 172–186.

[67] Lahbib Y Abidli S, Chiffoleau JF, Averty B, El Menif NT (2010) Imposex and butyltin concentrations in snails from the lagoon of Bizerta (Northern Tunisia). Mar Biol Res 6: 600–607.

[68] Vasconcelos P, Gaspar MB, Joaquim S, Matias D, Castro M (2004) Spawning of *Hexaplex (Trunculariopsis) trunculus* (Gastropoda: Muricidae) in the laboratory: description of spawning behaviour, egg masses, embryonic development, hatchling and juvenile growth rates. Invertebr Reprod Dev 46: 125–138.

[69] Schifano G (1983) Allometric growth as influenced by environmental temperature in *Monodonta turbinata* shells. Palaeogeogr Palaeocl 44: 215–222.

[70] Desai BN (1966) The biology of *Monodonta lineata* (Da Costa). J Mollus Stud 37: 1–17.

[71] Riesgo A, Maldonado M (2008) Differences in reproductive timing among sponges sharing habitat and thermal regime. Invertebr Biol 127: 357–367.

[72] Hayward P, Ryland J (1995) Handbook of the marine fauna of North-West Europe Oxford University Press.

[73] Brown SK, Roughgarden J (1985) Growth, morphology, and laboratory culture of larvae of *Balanus glandula* (Cirripedia, Thoracica). J Crustacean Biol 5: 574–590.

[74] Crisp DJ, Patel BS (1960) The moulting cycle in *Balanus balanoides* L. Biol Bull 118: 31–47.

