# Supplementary figures and images for: Genetic Structuring across Marine Biogeographic Boundaries in Rocky Shore Invertebrates
Source: PLoS One. 2014 Jul 1;9(7):e101135. doi: 10.1371/journal.pone.0101135 (PMC4077735; doi:10.1371/journal.pone.0101135)

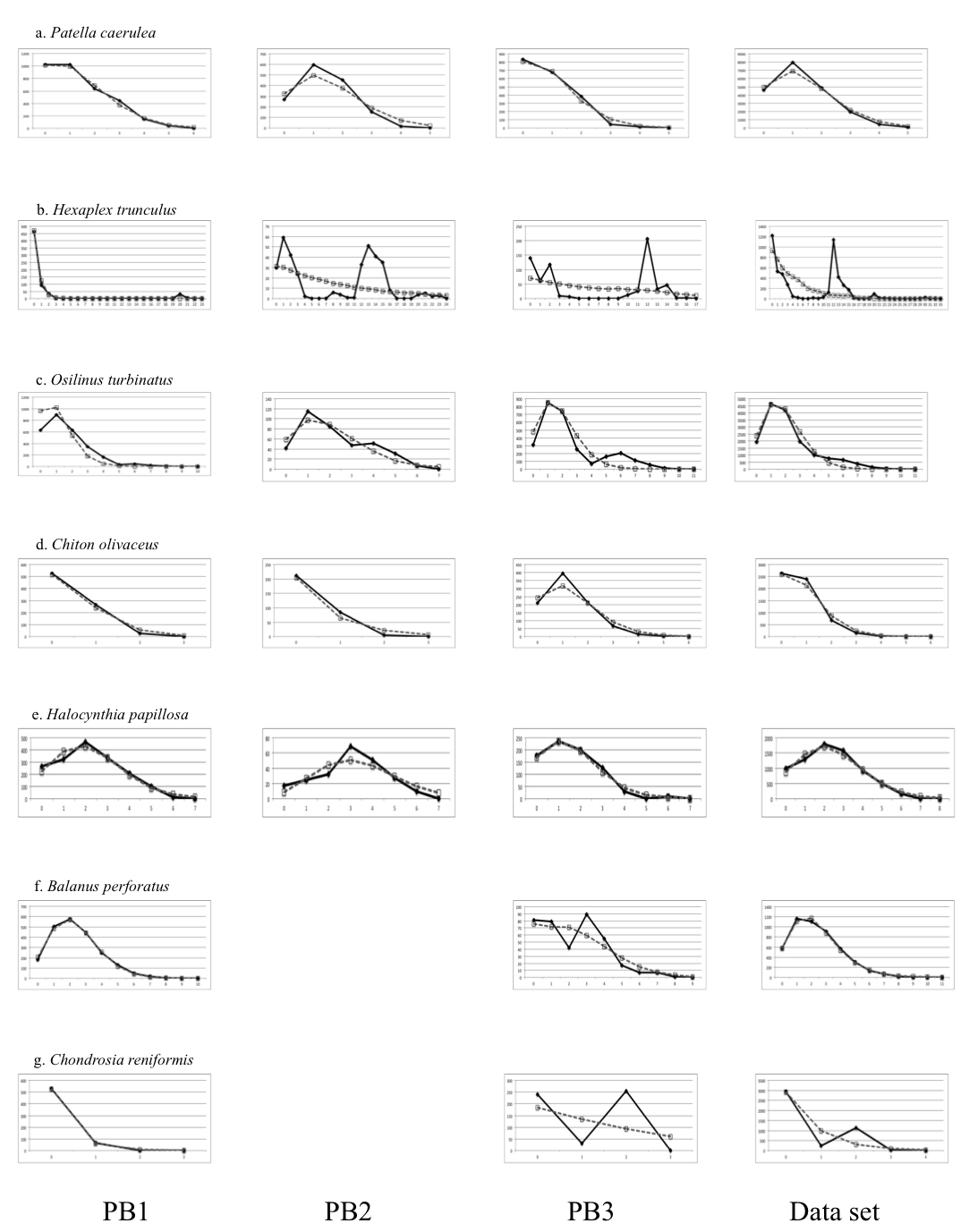

Supplement: Figure S1 — Mismatch distributions at each Location and for the whole data set for each of the seven species. Grey lines represent the expected and black lines the observed distribution. (TIF) [file pone.0101135.s001.tif]

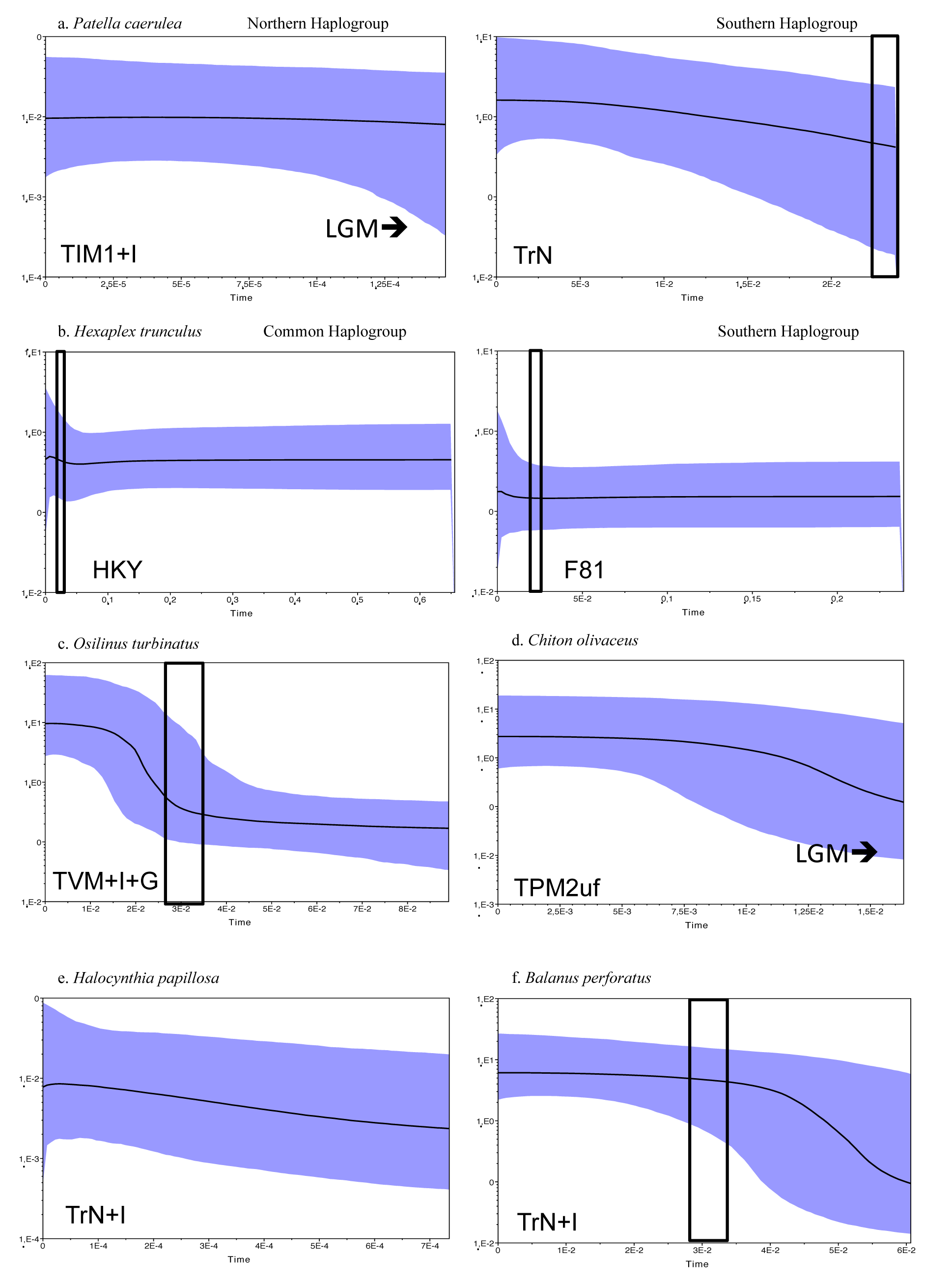

Supplement: Figure S2 — Bayesian Skyline Plots for the species showing signs of expansion according to mismatch distribution or neutrality tests. Patella caerulea and Hexaplex trunculus plots are calculated for each haplogroup. When shown, the black square indicates the date of the Last Glacial Maximum (LGM). The most likely evolutionary model selected by jModelTest is indicated in each plot. (TIF) [file pone.0101135.s002.tif]
