# Supplementary material for: Genetic Structuring across Marine Biogeographic Boundaries in Rocky Shore Invertebrates
Source: PLoS One. 2014 Jul 1;9(7):e101135. doi: 10.1371/journal.pone.0101135 (PMC4077735; doi:10.1371/journal.pone.0101135)
Supplement: Table S1 — Specific primers. Names, sequence, amplified fragment length, and optimal annealing temperature for each of the seven pairs of primers used. (DOCX) [file pone.0101135.s003.docx]

| **Table S1.** Names, sequence, amplified fragment length, and optimal annealing temperature for each of the seven pairs of primers used. | | | | |
| --- | --- | --- | --- | --- |
| Species | Primer name | Sequence 5’-3’ | Fragment length(bp) | Anneal. temp(**°**C) |
| *Patella caerulea* | LCO1490 | GGTCAACAAATCATAAAGATATTGG | 467 | 48 |
|  | HCO2198 | TAAACTTCAGGGTGACCAAAAAATCA |  |  |
| *Hexaplex trunculus* | HT-Fwd | ATATGGTCAGGGCTTGTTGG | 523 | 52 |
|  | HT-Rev | GGATCAAAGAACGCCGTATT |  |  |
| *Osilinus turbinatus* | OT-Fwd | CTCGTAGGAACCGCTCTCAG | 526 | 50 |
|  | OT-Rev | CTGCTGGGTCGAAGAAAGAG |  |  |
| *Chiton olivaceus* | ChO-Fwd | AAATAGGATCCCCTCCTCCA | 503 | 48 |
|  | ChO-Rev | CCAGGGGCTCTATTAGGTGA |  |  |
| *Halocynthia papillosa* | HP-Fwd | TTGTTTGGTGTTTGGTCTGG | 371 | 48 |
|  | HP-Rev | GCAGCTGCCAATACTGGTAAA |  |  |
| *Balanus perforatus* | BP-Fwd | ACGCTTCACACGAAAAGAGG | 442 | 52 |
|  | BP-Rev | ATTTTTGGAGCTTGGTCAGC |  |  |
| *Chondrosia reniformis* | ChR-Fwd | ACGGCCGTTTTACTCCTTCT | 428 | 48 |
|  | ChR-Rev | ACCGGTTATTTTCCCGAATC |  |  |
