# Supplementary material for: Genetic Structuring across Marine Biogeographic Boundaries in Rocky Shore Invertebrates
Source: PLoS One. 2014 Jul 1;9(7):e101135. doi: 10.1371/journal.pone.0101135 (PMC4077735; doi:10.1371/journal.pone.0101135)
Supplement: Table S2 — Previously published sequences. Accession numbers, geographical origin and identification with the haplotypes found in this study, as named in Figure 3, of sequences retrieved from Genbank. (DOCX) [file pone.0101135.s004.docx]

| **Table S2.** Accession numbers, geographical origin and identification with the haplotypes found in this study as named in Figure 3, of sequences retrieved from Genbank. | | | |
| --- | --- | --- | --- |
| Species | Accession number | Location | Haplotype in Figure 3 |
| *Patella caerulea* | GQ469862 | Ceuta, Spain | H_1 |
|  | GQ469863 | Cadiz, Spain | H_1 |
|  | GQ469864-GQ469867 | Sardinia, Italy | H_11(x2), H4(x2) |
|  | GQ469868 | Tuscany, Italy | H_1 |
|  | GQ469869 | Trieste, Italy | none |
|  | DQ089583 | Byzert, Tunisia | H_11 |
|  | DQ089584 | Byzert, Tunisia | H_4 |
|  | DQ089585 | Valencia, Spain | H_4 |
|  | DQ089586 | Valencia, Spain | H_1 |
|  | DQ089587 | Greece | H_1 |
|  | AB201519-AB201523 | West AOF, Spain | H_4(x3), H_17, H_26 |
|  | AB201524 | Chafarinas Is, Morocco | H_4 |
|  | AB201525 | West AOF, Spain | none |
|  | JN105784-JN105788 | Sardinia, Italy | H_4, H_8 |
|  | JN105789 | Ceuta, Spain | none |
|  | JN105790-JN105792 | Sardinia | H_4(x2), H_11 |
|  | JN105793 | Lampedusa, Italy | H_4 |
|  | JN105794 | Lampedusa, Italy | H_1 |
|  | JN105795 | Trieste, Italy | none |
|  | JN105796 | Trieste, Italy | H_8 |
| *Osilinus turbinatus* | JN686340 | South East Spain | H_4 |
|  | JN686341 | Cyprus | None, related to H_1 |
|  | JN686342 | Cyprus | H_9 |
|  | JN686343 | South East Spain | H_24 |
|  | JN686344 | Croatia | H_9 |
|  | JN686345 | Turkey | H_9 |
|  | JN686346 | South East Spain | H_24 |
|  | JN686347 | South East Spain | None, related to H_4 |
|  | JN686348 | Sardinia, Italy | H_4 |
|  | JN686349 | Sardinia, Italy | H_4 |
| *Hexaplex trunculus* | AM712604-AM712613 | South Portugal | H_4 (x9), 2 related to H_4 |
| *Chondrosia reniformis* | AM076986 | Israel | H_2 |
| *Chiton olivaceus* | AY377716 | North East Spain | H_1 |
| *Halocynthia papillosa* | AY600981 | North Western Mediterranean | H_12 |
|  | FJ528606-FJ528609 |  | H_13(x2), H_18(x2) |
|  | AY116603 |  | H_12 |
