# Supplementary material for: Genetic Structuring across Marine Biogeographic Boundaries in Rocky Shore Invertebrates
Source: PLoS One. 2014 Jul 1;9(7):e101135. doi: 10.1371/journal.pone.0101135 (PMC4077735; doi:10.1371/journal.pone.0101135)
Supplement: Table S3 — Analyses of Molecular Variance. Results of the Amova on the groups corresponding to the three Locations. (DOCX) [file pone.0101135.s005.docx]

| **Table S3.** Results of the Amova on the groups corresponding to the three Locations. | | | | | | | |
| --- | --- | --- | --- | --- | --- | --- | --- |
| Source of  variation | d.f. | Sum of squares | Variance components | Percentage of variation | Fixation Indices | p-values |  |
| ***a.Patella caerulea*** | | | | | | |  |
| Among groups | 2 | 12.153 | 0.07358 Va | 10.90 | FCT : 0.10900 | 0.132 |  |
| Among populations within groups | 3 | 3.737 | 0.02001 Vb | 2.96 | FST : 0.13864 | 0.000 |  |
| Within populations | 194 | 112.810 | 0.58149 Vc | 86.14 | FSC : 0.03327 | 0.006 |  |
| Total | 199 | 128.700 | 0.67509 |  |  |  |  |
| ***b. Hexaplex trunculus*** | | | | | | |  |
| Among groups | 2 | 89.900 | 0.84166 Va | 21.74 | FCT : 0.21737 | 0.271 |  |
| Among populations within groups | 3 | 47.930 | 0.86866 Vb | 22.43 | FST : 0.44171 | 0.000 |  |
| Within populations | 94 | 203.200 | 2.16170 Vc | 55.83 | FSC : 0.28665 | 0.000 |  |
| Total | 99 | 341.030 | 387.203 |  |  |  |  |
| ***c. Osilinus turbinatus*** | | | | | | |  |
| Among groups | 2 | 9.653 | 0.06517 Va | 7.29 | FCT : 0.07289 | 0.067 |  |
| Among populations within groups | 3 | 4.099 | 0.01996 Vb | 2.23 | FST : 0.09521 | 0.000 |  |
| Within populations | 166 | 134.295 | 0.80900 Vc | 90.48 | FSC : 0.02408 | 0.044 |  |
| Total | 171 | 148.047 | 0.89414 |  |  |  |  |
| ***d. Chondrosia reniformis*** | | | | | | |  |
| Among groups | 2 | 9.495 | 0.15109 Va | 43.42 | FCT : 0.43417 | 0.198 |  |
| Among populations within groups | 3 | 0.234 | -0.00915 Vb | -2.63 | FST : 0.40788 | 0.000 |  |
| Within populations | 88 | 18.133 | 0.20605 Vc | 59.21 | FSC : -0.04646 | 0.589 |  |
| Total | 93 | 27.862 | 0.34799 |  |  |  |  |
| ***e. Chiton olivaceus*** | | | | | |  |  |
| Among groups | 2 | 2.866 | 0.00973 Va | 2.57 | FCT : 0.02569 | 0.270 |  |
| Among populations within groups | 3 | 3.088 | 0.04074 Vb | 10.75 | FST : 0.13321 | 0.000 |  |
| Within populations | 103 | 33.826 | 0.32841 Vc | 86.68 | FSC : 0.11036 | 0.001 |  |
| Total | 108 | 39.780 | 0.37888 |  |  |  |  |
| ***f. Halocynthia papillosa*** | | | | | | |  |
| Among groups | 2 | 15.678 | 0.16665 Va | 13.56 | FCT : 0.13561 | 0.064 |  |
| Among populations within groups | 3 | 4.810 | 0.02891 Vb | 2.35 | FST : 0.15914 | 0.000 |  |
| Within populations | 115 | 118.834 | 1.03334 Vc | 84.09 | FSC : 0.02722 | 0.151 |  |
| Total | 120 | 139.322 | 122.891 |  |  |  |  |
| ***g. Balanus perforatus*** | | | | | |  |  |
| Among groups | 1 | 0.738 | -0.00019 Va | -0.02 | FCT : -0.00017 | 1 |  |
| Among populations within groups | 2 | 1.371 | -0.02095 Vb | -1.83 | FST : -0.01843 | 0.990 |  |
| Within populations | 90 | 105.157 | 1.16841 Vc | 101.84 | FSC : -0.01826 | 0.964 |  |
| Total | 93 | 107.266 | 114.727 |  |  |  |  |
