# Supplementary material for: Genetic Structuring across Marine Biogeographic Boundaries in Rocky Shore Invertebrates
Source: PLoS One. 2014 Jul 1;9(7):e101135. doi: 10.1371/journal.pone.0101135 (PMC4077735; doi:10.1371/journal.pone.0101135)
Supplement: Table S4 — Demographic history analyses. Fit to sudden expansion model and neutrality tests. (DOCX) [file pone.0101135.s006.docx]

| **Table S4.** Fit to sudden expansion model and neutrality tests | | | | | | | |
| --- | --- | --- | --- | --- | --- | --- | --- |
| Site | A | B | C | D | E | F | Total |
| ***a. Patella caerulea*** | | | | | | | |
| **Mismatch distribution** | | | | | | |  |
| Model (SSD) p-value | 0.92000 | 0.95000 | 0.08000 | 0.25000 | 0.81000 | 0.64000 | 0.03000 |
| **Neutrality tests** |  |  |  |  |  |  |  |
| Tajima's D | -1.62314 | -1.85089 | -1.17183 | -1.45861 | -0.97904 | -1.11484 | -2.20189 |
| Tajima's D p-value | 0.03370 | 0.01160 | 0.12270 | 0.05900 | 0.18350 | 0.14260 | 0.00030 |
| Fu’s FS | -7.07734 | -8.38258 | -8.08678 | -5.13118 | -1.18779 | -1.89531 | -28.51326 |
| Fu’s FS p-value | 0.00000 | 0.00000 | 0.00000 | 0.00040 | 0.20870 | 0.08350 | 0.00000 |
| ***b. Hexaplex trunculus*** | | | | | | |  |
| **Mismatch distribution** | | | | | | |  |
| Model (SSD) p-value | 0.33000 | 0.24000 | 0.41000 | 0.49000 | 0.07000 | 0.02000 | 0.34000 |
| **Neutrality tests** |  |  |  |  |  |  |  |
| Tajima's D | -2.55327 | -1.14053 | -1.51480 | -0.55326 | 1.42714 | 1.41819 | -0.91070 |
| Tajima's D p-value | 0.00000 | 0.16300 | 0.05400 | 0.35600 | 0.94200 | 0.94100 | 0.18440 |
| Fu’s FS | 0.98022 | -0.47566 | 1.12266 | -0.25844 | 3.38049 | 1.41390 | -3.67242 |
| Fu’s FS p-value | 0.76000 | 0.11600 | 0.73900 | 0.46000 | 0.93100 | 0.76900 | 0.17150 |
| ***c. Osilinus turbinatus*** |  |  |  |  |  |  |  |
| **Mismatch distribution** | | | | | | |  |
| Model (SSD) p-value | 0.85220 | 0.92520 | 0.12410 | 0.06820 | 0.84940 | 0.07980 | 0.03000 |
| **Neutrality tests** |  |  |  |  |  |  |  |
| Tajima's D | -1.40186 | -1.74174 | -0.53368 | -1.32986 | -0.99713 | -0.60957 | -1.91142 |
| Tajima's D p-value | 0.07210 | 0.02000 | 0.32900 | 0.08210 | 0.16980 | 0.29120 | 0.00340 |
| Fu’s FS | -11.50330 | -13.75772 | -4.00470 | -5.98387 | -2.02859 | -5.59118 | -27.12273 |
| Fu’s FS p-value | 0.00000 | 0.00000 | 0.01840 | 0.00060 | 0.04400 | 0.00080 | 0.00000 |
| ***d. Chondrosia reniformis*** |  |  |  |  |  |  |  |
| **Mismatch distribution** | | | | | | |  |
| Model (SSD) p-value | 0.26600 | 0.10900 | 0.00000 | 0.00000 | 0.13700 | 0.13500 | 0.08900 |
| **Neutrality tests** |  |  |  |  |  |  |  |
| Tajima's D | -1.15753 | -1.11173 | 0.00000 | 0.00000 | 1.66480 | 2.06053 | -0.46860 |
| Tajima's D p-value | 0.14340 | 0.18490 | 100.000 | 100.000 | 0.96980 | 0.99170 | 0.37480 |
| Fu’s FS | -1.06131 | -0.33931 | 0.00000 | 0.00000 | 0.69375 | 3.17767 | -1.00609 |
| Fu’s FS p-value | 0.00490 | 0.15100 | N.A. | N.A. | 0.60760 | 0.91970 | 0.28610 |
| ***e. Chiton olivaceus*** | | | | | | |  |
| **Mismatch distribution** | | | | | | |  |
| Model (SSD) p-value | 0.37400 | 0.48600 | 0.09400 | 0.49600 | 0.19100 | 0.36800 | 0,231 |
| **Neutrality tests** |  |  |  |  |  |  |  |
| Tajima's D | -2.08749 | -1.49796 | -1.15945 | -1.56222 | -1.42890 | -1.17523 | -2.26296 |
| Tajima's D p-value | 0.00300 | 0.04800 | 0.15200 | 0.04600 | 0.07500 | 0.10800 | 0.00042 |
| Fu’s FS | -6.13807 | -1.61464 | -0.64899 | -1.96374 | -3.79119 | -3.65222 | -23.92137 |
| Fu’s FS p-value | 0.00000 | 0.01600 | 0.11600 | 0.00800 | 0.00600 | 0.00700 | 0.00000 |
| ***f. Halocynthia papillosa*** |  |  |  |  |  |  |  |
| **Mismatch distribution** | | | | | | |  |
| Model (SSD) p-value | 0.14200 | 0.69000 | 0.47300 | 0.51500 | 0.59000 | 0.63200 | 0.73000 |
| **Neutrality tests** |  |  |  |  |  |  |  |
| Tajima's D | 0.67641 | 1.04900 | 1.19457 | 1.10026 | -0.06781 | 0.17848 | 1.22466 |
| Tajima's D p-value | 0.77410 | 0.86610 | 0.88990 | 0.87380 | 0.50480 | 0.62370 | 0.89760 |
| Fu’s FS | -1.90966 | -1.16773 | 0.10844 | -4.06043 | -0.05164 | 0.05479 | -7.10080 |
| Fu’s FS p-value | 0.16140 | 0.27660 | 0.51170 | 0.00640 | 0.48360 | 0.51940 | 0.01220 |
| ***g. Balanus perforatus*** | | | | | | |  |
| **Mismatch distribution** | | | | | | |  |
| Model (SSD) p-value | 0.98000 | 0.45000 |  |  | 0.25000 | 0.52000 | 0.86480 |
| **Neutrality tests** |  |  |  |  |  |  |  |
| Tajima's D | -2.00403 | -1.95131 |  |  | -1.16290 | -1.59619 | -2.34248 |
| Tajima's D p-value | 0.00500 | 0.00600 |  |  | 0.13200 | 0.04200 | 0.00030 |
| Fu’s FS | -15.48994 | -18.75185 |  |  | -1.60653 | -3.13979 | -27.06793 |
| Fu’s FS p-value | 0.00000 | 0.00000 |  |  | 0.09700 | 0.03500 | 0.00000 |
